# How to Take HIV Antiretroviral Medications on Time without a Watch in Rural Uganda

**DOI:** 10.1371/journal.pmed.0030161

**Published:** 2006-03-28

**Authors:** Marissa Maier, Mwebesa Bwana, Nneka Emenyonu, Larry Pepper, David R Bangsberg

**Affiliations:** **1**University of California San FranciscoSan Francisco, CaliforniaUnited States of America; **2**Mbarara University of Science and TechnologyMbararaUganda

Castro has advocated that adherence to HIV antiretroviral therapy should be understood within a patient's clinical and social context [ 1]. Over 90% of worldwide HIV infection occurs in resource-limited settings [ 2]. Some have suggested that individuals living in extreme poverty may have difficulties with adherence to medication [ 3], including Andrew Natisios, who said Africans “don't know what Western time is” [ 4]. While recent reports suggest that adherence to HIV antiretroviral therapy in resource-limited settings may be as good as or better than resource-rich settings [ 5–7], the question remains: how do people take medications on time without a watch?

In rural western Uganda, there is, for example, a 40-year-old man who is HIV-positive, has no education, and works as a farmer. He lives with his brother, sister-in-law, and three nieces in a three-room, mud-walled house without electricity. He owns a lantern, a bed, a sofa, a bike, and a radio, but does not own a watch. He was diagnosed with HIV in April 2005 and started generic D4T/3TC/NVP (Triomune) four months after developing disseminated herpes zoster and Kaposi sarcoma with a CD4 count of 151. His adherence was measured with an electronic medication monitor that records a date-time stamp in flash memory each time the pill container is opened. Over the 89 days of monitored treatment, he had 98.9% adherence by electronic monitor and took 90% of prescribed doses within ten minutes of 7:20 a.m. and within 17 minutes of 7:20 p.m. When asked how he knew when to take his dose, he said that he knows it is time to take his medications by “listening to Radio West's ‘News and Announcements’ every morning and evening.” [Fig pmed-0030161-g001] [Fig pmed-0030161-g002]

**Figure pmed-0030161-g001:**
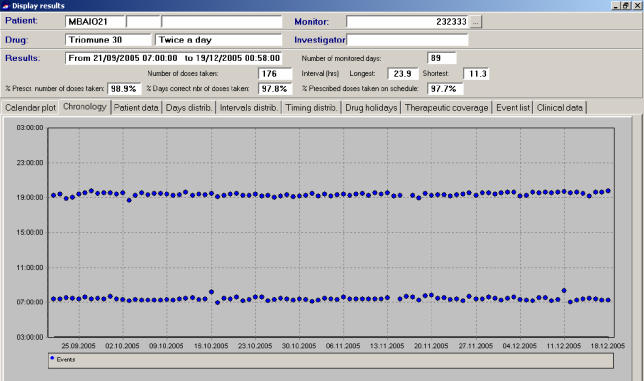
Electronic medication monitor adherence record of time of bottle openings for morning and evening doses

**Figure pmed-0030161-g002:**
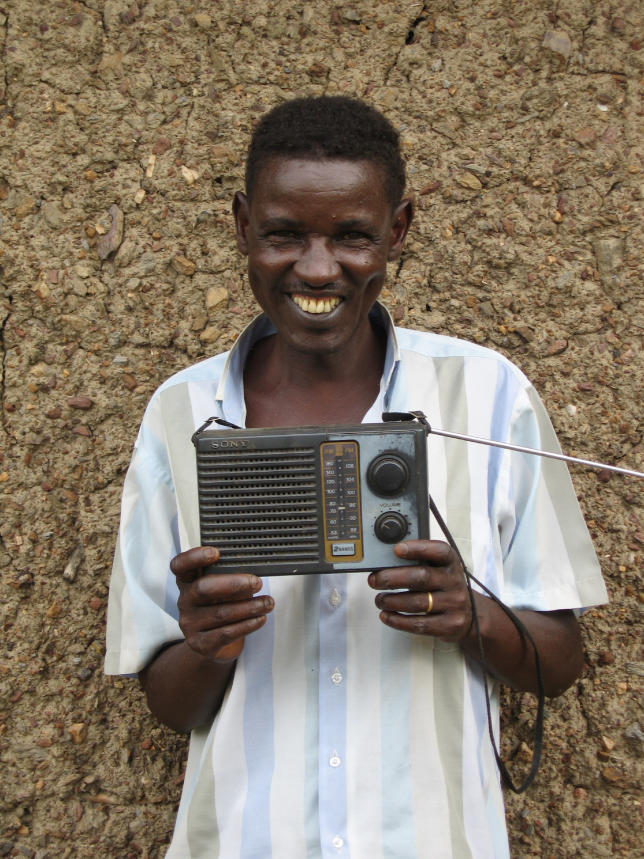
Patient with his radio

While population levels of adherence will likely drop as treatment access expands and people begin to experience toxicities of long-term therapy, he is an example of how patients can have precise, if not perfect, adherence with creative solutions in a resource-limited setting.
